# Spaceflight-Induced Gene Expression Profiles in the Mouse Brain Are Attenuated by Treatment with the Antioxidant BuOE

**DOI:** 10.3390/ijms241713569

**Published:** 2023-09-01

**Authors:** Isaac Kremsky, Samir Ali, Seta Stanbouly, Jacob Holley, Stephen Justinen, Michael Pecaut, James Crapo, Xiaowen Mao

**Affiliations:** 1 Department of Basic Sciences, Division of Biomedical Engineering Sciences (BMES), Loma Linda University School of Medicine, Loma Linda, CA 92350, USA; ikremsky@llu.edu (I.K.); samirali@llu.edu (S.A.); sstanbouly@llu.edu (S.S.); jholley@llu.edu (J.H.); sjustinen@students.llu.edu (S.J.); mpecaut@llu.edu (M.P.); 2 Center for Genomics, Loma Linda University School of Medicine, Loma Linda, CA 92350, USA; 3 Department of Medicine, Division of Pulmonary, Critical Care & Sleep Medicine, National Jewish Health, University of Colorado Denver, Denver, CO 80206, USA; james.crapo@gmail.com

**Keywords:** spaceflight, brain, digital spatial profiling, gene expression, regional difference

## Abstract

The demands of deep space pose a health risk to the central nervous system that has long been a concern when sending humans to space. While little is known about how spaceflight affects transcription spatially in the brain, a greater understanding of this process has the potential to aid strategies that mitigate the effects of spaceflight on the brain. Therefore, we performed GeoMx Digital Spatial Profiling of mouse brains subjected to either spaceflight or grounded controls. Four brain regions were selected: Cortex, Frontal Cortex, Corunu Ammonis I, and Dentate Gyrus. Antioxidants have emerged as a potential means of attenuating the effects of spaceflight, so we treated a subset of the mice with a superoxide dismutase mimic, MnTnBuOE-2-PyP 5+ (BuOE). Our analysis revealed hundreds of differentially expressed genes due to spaceflight in each of the four brain regions. Both common and region-specific transcriptomic responses were observed. Metabolic pathways and pathways sensitive to oxidative stress were enriched in the four brain regions due to spaceflight. These findings enhance our understanding of brain regional variation in susceptibility to spaceflight conditions. BuOE reduced the transcriptomic effects of spaceflight at a large number of genes, suggesting that this compound may attenuate oxidative stress-induced brain damage caused by the spaceflight environment.

## 1. Introduction

Radiation exposure and other stressors experienced during extended deep-space missions have the potential to compromise the function of many bodily systems [1,2,3]. The health risks of spaceflight-induced damage related to the central nervous system (CNS), such as cognitive impairment and neurodegenerative effects, have long been a concern. Animal studies have shown that memory, cognition, motor activity, and other neural functions can be affected under stressful conditions that include both radiation exposure and the spaceflight environment [4,5,6,7,8].

Normal brain function relies on a diverse set of differentiated cell types, including neurons, glia, and vasculature in different regions. There have been, however, relatively few studies on the effects of spaceflight stress on specific brain regions. One study found that spaceflight conditions induced distinct protein expression changes in different regions of the mouse brain [9]. Regional differences were also documented following simulated microgravity in human brain gray and white matter [10]. Our previous flight study [11] demonstrated that exposure to the spaceflight environment induces significant changes in protein expression related to neuronal structure and metabolic function, and there were distinct changes in protein expression in grey versus white matter.

One brain region that is of immense interest in the context of spaceflight is the cortex (CT). It plays a key role in sensory and motor function and is closely associated with locomotion, learning, memory, and coordination [12]. Spaceflight and ground simulation studies have given evidence that microgravity conditions negatively affect sensorimotor and behavioral performance, and the functional architecture of the human brain [13,14,15,16], all of which are associated with cortical activity. These significant neurochemical changes were also observed in the rat prefrontal cortex by heavy charged particle irradiation [17]. The frontal cortex (FCT) plays a role in memory, attention, judgment, consciousness, and behavior [18]. Pathophysiological changes in the FCT are associated with depression and anxiety [19].

Another brain region that is critical to understand in the context of spaceflight is the hippocampus, a complex brain structure embedded deep in the temporal lobe. It plays a major role in learning and memory [20]. Ground-based analog studies have found that simulated microgravity influences cognitive function, with increased anxiety and depression-like behaviors that were associated with hippocampal activities [21]. In addition, significantly altered expression of many proteins that relate to metabolism and structure in the hippocampus has been observed to occur under simulated microgravity [22,23]. Space radiation studies have revealed cognitive detriment and changes in morphology in the hippocampal dentate gyrus (DG) and Cornu Ammonis 1 (CA) regions following oxygen-particle irradiation [24]. The DG is located in the deep region of the hippocampus and has been shown to play an important role in pattern separation and associative memory [25]. The CA contains pyramidal cells with a vast network of interneurons; its function is related to memory and consciousness [25].

Spaceflight conditions are associated with oxidative stress [26], which contributes to cellular damage in a variety of tissues. MnTnBuOE-2-PyP^5+^ (BuOE), a manganese porphyrin superoxide dismutase (SOD) mimic, also termed BMX-001, is remarkably beneficial in many animal models of oxidative stress injury [27]. One study demonstrated that BuOE can reduce oxidative stress damage to the brain caused by radiation exposure [28]. Our recent study [29] showed that BuOE treatment during spaceflight significantly reduces the immunoreactivity of the oxidative stress biomarker 4-hydroxynonena (4-HNE) in the retina. This suggests that BuOE is effective in alleviating stress responses to spaceflight [29]. Our present study will test whether BuOE treatment during spaceflight can attenuate spaceflight-induced alterations in gene transcription in specific brain regions.

Despite broad interest in understanding region-specific stress responses, characterizing these changes has been a challenge. More recently, spatial transcriptomics technology has been developed, which allows us to simultaneously quantify gene expression levels and their spatial distribution within tissue sections. This method can help to better understand biological responses as well as disease development [30,31]. In this study, we took advantage of this technology by using the Nanostring GeoMx^®^ digital spatial profiling (DSP) platform [32] to investigate spaceflight-induced changes in gene expression profiles in mouse CT, FCT, and hippocampal DG and CA regions, both with and without BuOE treatment. Our unique spatial sequencing data may, for the first time, provide insights into the organization and response of specific brain regions to the spaceflight environment, potentially improving risk assessment of long-term space travel.

## 2. Results

### 2.1. Gene Expression Profiling of CA, DG, CT, and FCT Samples Subjected to Spaceflight

**Figure 1:**
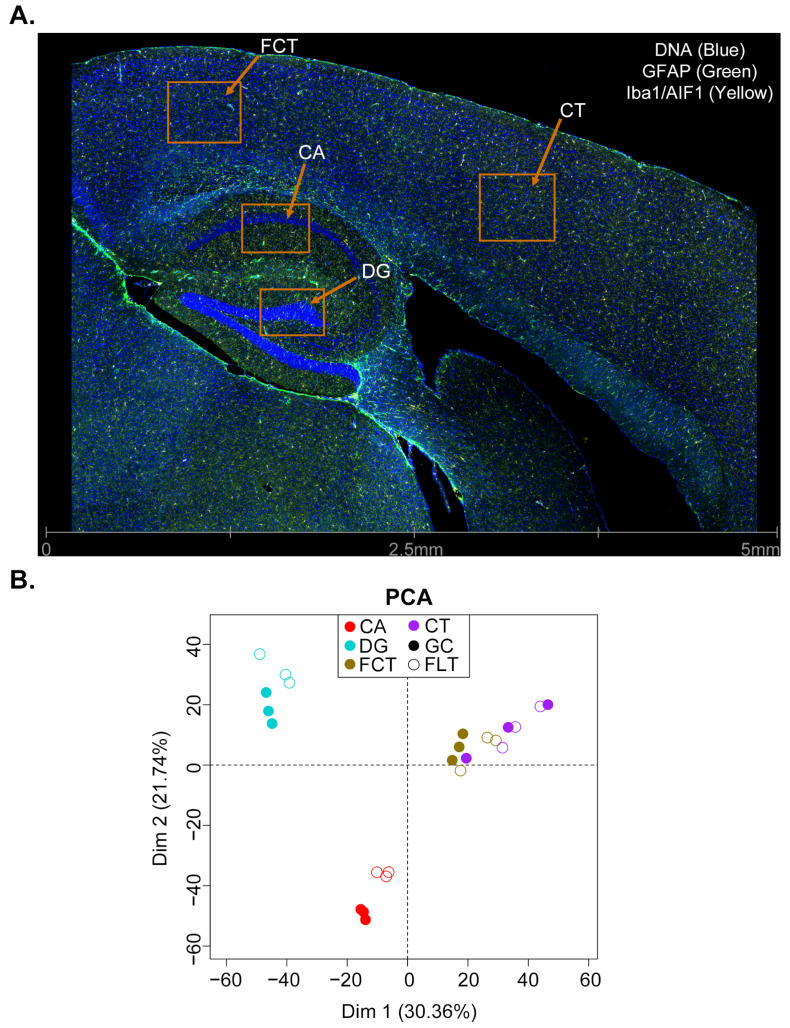
DSP of the mouse brain under spaceflight and grounded control conditions. (**A**), A representative image (FLT_SAL_2|005, 006, 007, and 008) of the brain regions profiled by DSP in this study. (**B**) Principal components analysis of the SAL transcriptomic data from the indicated brain regions. Filled circles represent the GC data, while empty circles represent the FLT data.

We performed in situ RNA assays in the brains of mice subjected to spaceflight (FLT), as well as grounded controls (GC), using DSP. Half of the mice were treated with BuOE, while the other half were treated with saline (SAL). Four regions within the brain of each mouse were assayed: the CA, DG, CT, and FCT (Figure 1A). All samples achieved high sequencing saturation (Supplementary File S1: Figure S1A), and Q3 normalization was performed on the data (Supplementary File S1: Figure S1B) for all downstream analyses. Normalized count values of gene expression were highly correlated across replicates of the four samples (Supplementary File S1: Figure S1C–F). A principal components analysis (PCA) revealed that spaceflight had an effect on all four regions in SAL control animals, with CA and DG being impacted the most (Figure 1B).

**Figure 2:**
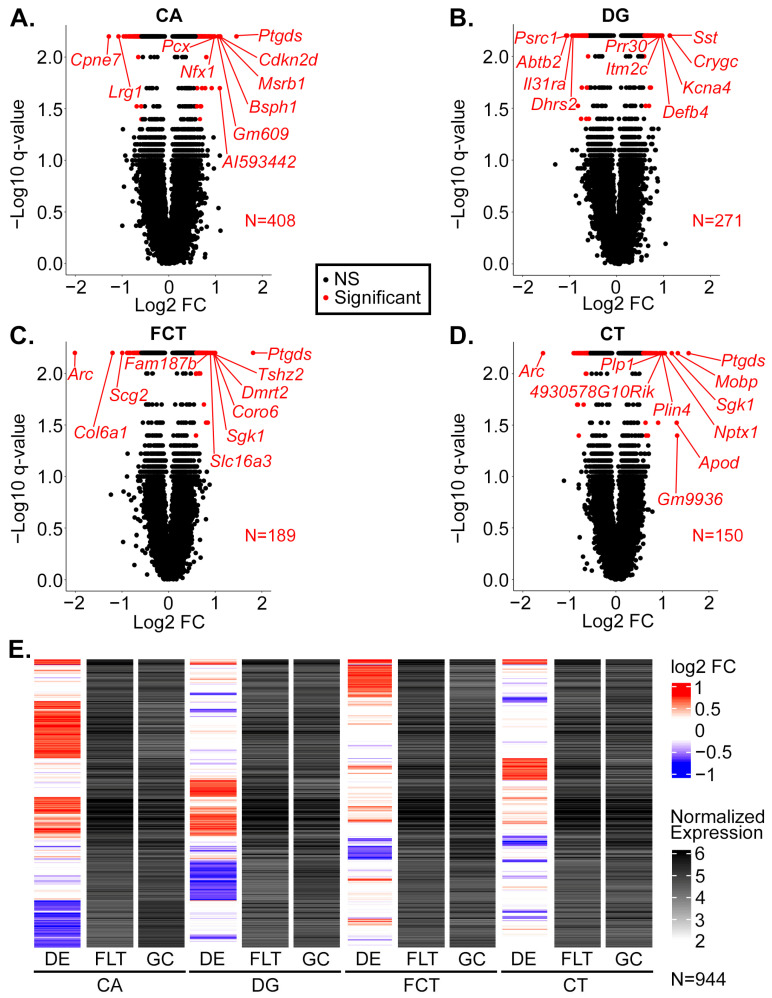
Differentially expressed genes due to spaceflight conditions. (**A**–**D**), Volcano plots summarizing the differential gene expression analysis of the indicated region, comparing FLT-SAL versus GC-SAL. The number of DEGs is indicated for each brain region. Gene symbols are shown for the top 10 DEGs by the magnitude of the log_2_ fold change (FC). (**E**), Heatmap of differential expression (DE) using log_2_ FC values of the FLT-SAL versus GC-SAL DEGs from the indicated regions, along with normalized gene expression values of the indicated samples and regions. Genes are ordered by hierarchical clustering of the log_2_ FC values.

We next performed a differential expression analysis (FLT versus GC) on the SAL data from each brain region and identified a total of 408 differentially expressed genes (DEGs) in CA, 271 in DG, 189 in FCT, and 150 in CT (Figure 2A–D). Each brain region largely had a unique set of DEGs, though a small number of DEGs were common to two or more regions (Figure 2E).

**Figure 3:**
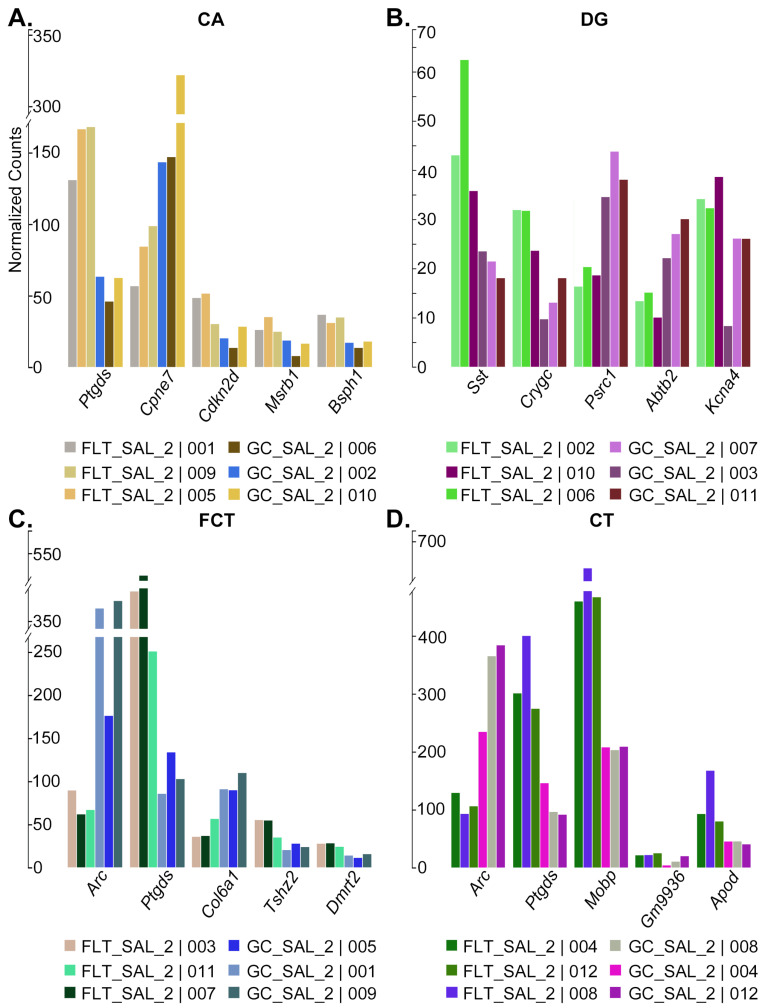
Top spaceflight DEGs. (**A**–**D**), Barplots showing the normalized expression values of the top 5 DEGs (FLT-SAL versus GC-SAL) by magnitude of log_2_ fold change.

The top five DEGs by magnitude of fold change are as follows: *Ptgds*, *Cpne7*, *Cdkn2d*, *Msrb1*, and *Bsph1* for CA (Figure 3A and Supplementary File S1: Figure S2A), *Sst*, *Crygc*, *Psrc1*, *Abtb2*, and *Kcna4* for DG (Figure 3B and Supplementary File S1: Figure S2B), *Arc*, *Ptgds*, *Col6a1*, *Tshz2*, and *Dmrt2* for FCT (Figure 3C and Supplementary File S1: Figure S2C), and *Arc*, *Ptgds*, *Mobp*, *Gm9936*, and *Apod* for CT (Figure 3D and Supplementary File S1: Figure S2D). Many of these top-changing genes play important roles in apoptosis, cell cycle, neuroinflammation, neurotransmission, myelination, and mitochondrial and metabolic stress response.

In order to determine whether spaceflight might accelerate certain disease processes in the brain, we compared the FLT-SAL vs. GC-SAL DEGs to curated lists of gene-disease associations. Alzheimer’s disease (AD) was the most frequent disease associated with the DEGs, and each brain region had at least one DEG with a known association with AD; in all, 22 of the GC-SAL vs. FLT-SAL DEGs have a known association with a neurological disease (Supplementary File S2). This raises the possibility that spaceflight stress may increase the risk of onset and/or progression of certain neurological disorders, such as AD.

**Figure 4:**
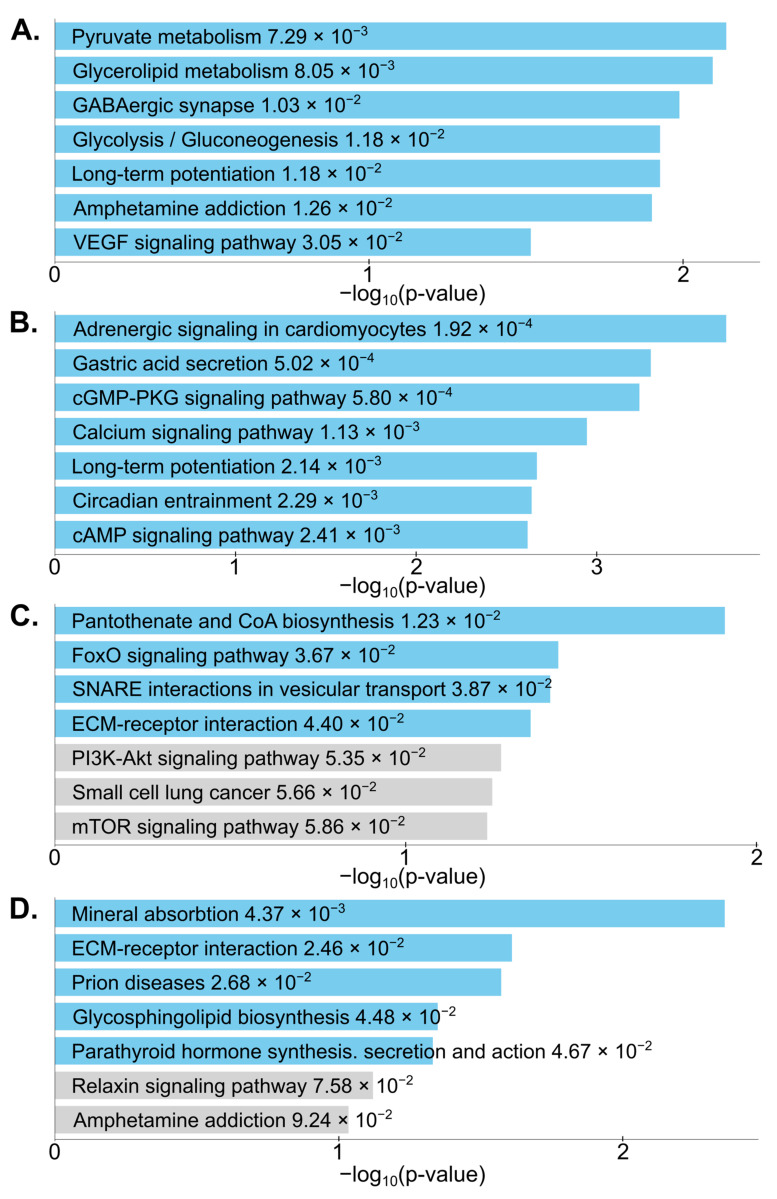
Pathway analysis. Bar plots showing the *p*-value for the 7 most statistically significant pathways containing FLT-SAL versus GC-SAL DEGs in (**A**): CA, (**B**): DG, (**C**): FCT, (**D**): CT.

Next, we performed a pathway analysis of the GC-SAL vs. FLT-SAL DEGs from each region independently. Importantly, each of the four brain regions had at least one pathway known to be affected by oxidative stress that was significantly enriched: VEGF signaling pathway in CA (Figure 4A), Calcium signaling pathway in DG (Figure 4B), FoxO signaling pathway in FCT (Figure 4C), and ECM-receptor interaction in CT (Figure 4D). In addition, a number of metabolic pathways were also significantly enriched, such as pyruvate metabolism and glycolysis/gluconeogenesis in CA, cAMP signaling pathway in DG, pantothenate and CoA biosynthesis in FCT, and glycosphingolipid biosynthesis in CT.

### 2.2. BuOE Attenuates Spaceflight-Induced Transcriptional Changes in the CA, DG, FCT, and CT

**Figure 5:**
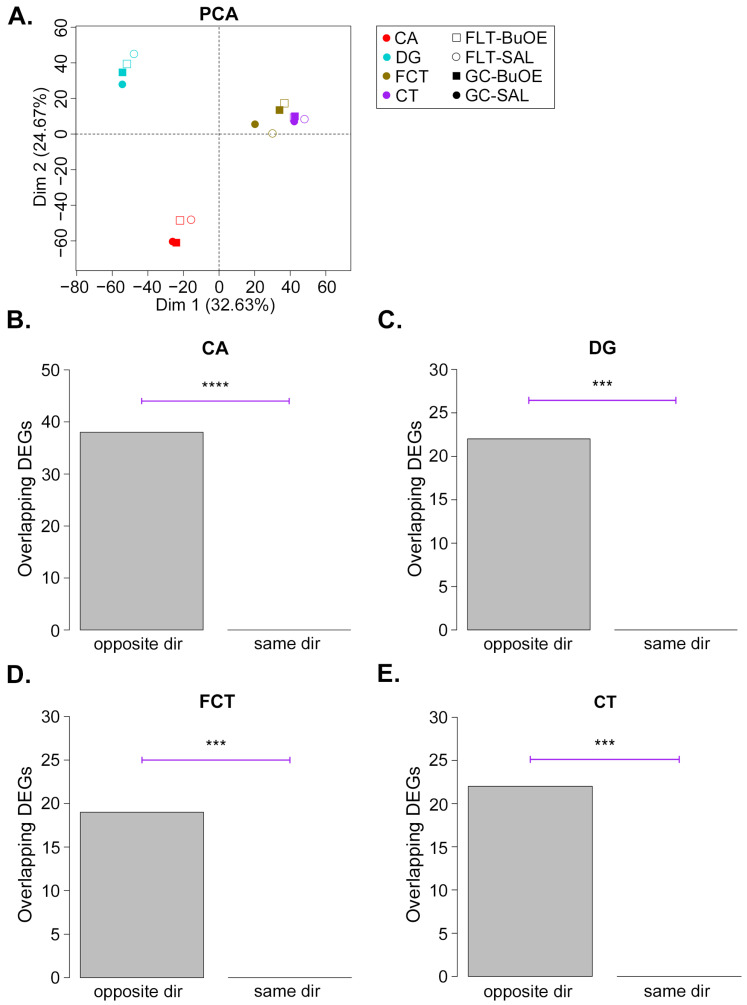
BuOE attenuates spaceflight-induced gene expression changes. (**A**), Principal components analysis of the indicated regions and treatment conditions. Each gene was averaged across replicates to obtain the PCA. Individual replicates are shown in the PCA in Supplementary File S1: Figure S3A. (**B**–**E**), Barplots comparing the number of spaceflight-induced DEGs (GC-SAL vs. FLT-SAL) that change in the opposite versus the same direction (dir) as BuOE-induced DEGs (FLT-SAL vs. FLT-BuOE). *p*-values were calculated by Fisher’s Exact test. *** *p* < 10^−5^; **** *p* < 10^−10^.

Finally, given that we observed a robust transcriptional response to spaceflight, we sought to determine if treatment with BuOE would attenuate these changes. A PCA of global transcription shows that in all brain regions examined except FCT, BuOE during spaceflight brought transcription levels closer to GC animals (Figure 5A and Supplementary File S1: Figure S3A), suggesting a partial attenuation of spaceflight transcriptional alterations by BuOE. Even for FCT, the distance between FLT-BuOE and GC-BuOE was smaller than the distance between FLT-SAL and GC-SAL, again suggesting BuOE results in a reduced transcriptional response due to spaceflight. We obtained BuOE-induced DEGs (FLT-SAL vs. FLT-BuOE) using the same criteria as for the spaceflight-induced DEGs (GC-SAL vs. FLT-SAL). We expected that if BuOE attenuates spaceflight-induced differential expression, then BuOE-induced DEGs should tend to change in the opposite direction as the spaceflight-induced DEGs. Indeed, for all brain regions, significantly more spaceflight-induced DEGs changed in the opposite direction as BuOE-induced DEGs (Figure 5B–E). We also performed a permutation test (see Section 4) in which genes were shuffled in order to randomly select up- and down-regulated BuOE-induced DEGs, and checked the overlaps with the spaceflight-induced DEGs. For all brain regions, the number of random overlaps after 1000 permutations was never as large as the number of actual overlaps in the opposite direction (Supplementary File S1: Figure S3B–E).

**Figure 6:**
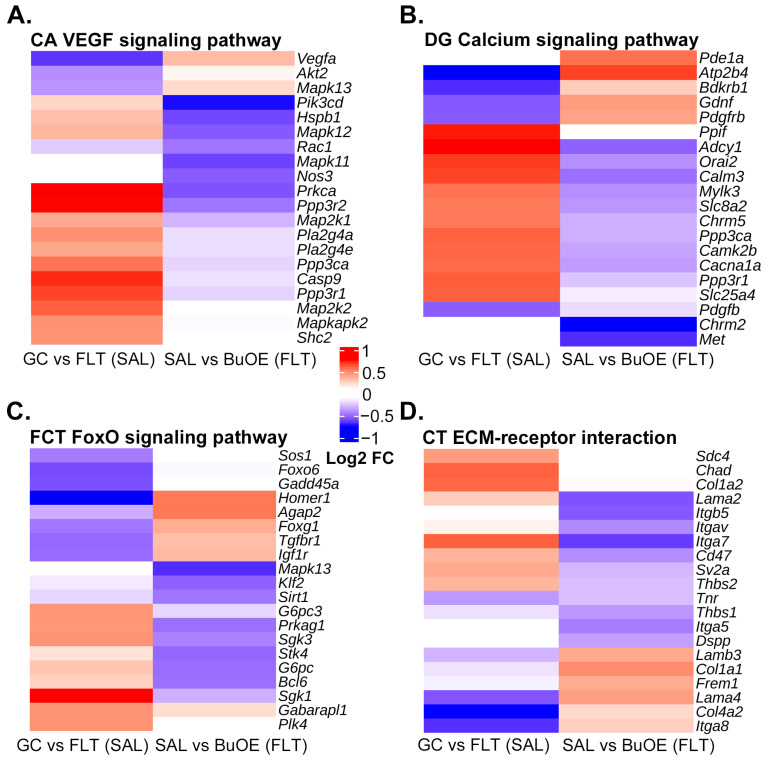
BuOE attenuation of spaceflight-induced gene expression changes of genes in oxidative stress–responsive pathways. (**A**–**D**), log2 fold change (FC) values of genes from the indicated comparisons, regions, and pathways. The top 20 genes by magnitude of log_2_ FC from either of the two comparisons are shown. Genes are ordered by hierarchical clustering. GC vs. FLT (SAL) = GC-SAL versus FLT-SAL; SAL vs. BuOE (FLT) = FLT-SAL versus FLT-BuOE.

At many of the spaceflight-induced DEGs, the direction of change in expression by BuOE during spaceflight, regardless of significance, tended to be in the opposite direction, and in many cases these changes were not induced by BuOE in GC mice (Supplementary File S1: Figure S4A), again suggesting a partial attenuation of spaceflight-induced transcriptional changes in the brain by BuOE. Of note, such an attenuation was observed for several genes in the significantly enriched pathways responsive to oxidative stress; e.g., VEGF signaling pathway in CA (Figure 6A), Calcium signaling pathway in DG (Figure 6B), FoxO signaling pathway in FCT (Figure 6C), and ECM-receptor interaction in CT (Figure 6D). Similarly, BuOE also attenuated spaceflight-induced changes in the expression of several genes associated with Alzheimer’s disease (Supplementary File S1: Figure S4B–E).

## 3. Discussion

In this study, we performed a differential expression analysis of spaceflight versus GC mice and identified 944 genes that were significantly altered by spaceflight in at least one of the four brain regions examined. Our results revealed common and region-specific gene expression changes in the brain due to spaceflight, with the most robust changes observed in the hippocampal CA 1 and DG regions, suggesting that these regions may be particularly vulnerable to spaceflight. The hippocampus is not only the center for learning and memory, it is a site for neurogenesis as well [33]. Numerous cell types in the hippocampus contribute to generating neurons important for encoding new memories, spatial learning, and cognitive flexibility [34]. Space radiation may damage hippocampal cells [35] and thus potentially suppress neurogenesis, leading to memory decline, anxiety, and depression [34]. Hippocampal neuronal circuitry also modulates and affects physiology and functional connectivity with more distant brain regions, including the prefrontal cortex [36,37,38].

In order to determine whether spaceflight might accelerate certain disease processes in the brain, we looked for known associations with neurological disease amidst the spaceflight-induced DEGs. Several of the DEGs have known associations with Alzheimer’s disease, as well as other neurological diseases. Previous studies have shown that space radiation contributes to amyloid pathologies, neuroinflammation, and cognitive function impairments resembling age-associated cognitive decline in animals [4,39,40]. Our results add to the growing body of evidence that space stressors could induce or accelerate neurodegenerative processes.

To obtain insights into the biological processes being affected in the brain by spaceflight, we performed pathway analyses on the spaceflight-induced DEGs. Interestingly, several pathways known to be altered by oxidative stress were significantly enriched in the four brain regions due to spaceflight: VEGF signaling [41] in CA, calcium signaling [42] in DG, FoxO signaling [43] in FCT, and ECM-receptor interaction [44] in CT. In addition, a number of metabolic pathways were among the most significantly enriched, consistent with numerous studies that have found alterations in metabolism and its regulation due to spaceflight-induced neuroendocrine and psychophysiological changes [45]. For example, a mouse study revealed an accumulation of lipids in the liver after spaceflight [46]. Studies also show that space stressors induce metabolic changes in mouse plasma and in the expression of metabolism-related genes [47]. Longitudinal metabolomic profiles revealed sex-specific perturbations in glucose and amino acid metabolism that result from the stressors of long-duration spaceflight [48]; therefore, follow-up studies on sexually dimorphic transcriptional responses to spaceflight in the brain may be warranted.

The Circadian Entrainment pathway was significantly altered by spaceflight in DG. This is consistent with our previous spaceflight genomic study [49], which showed spaceflight altered some genes associated with circadian rhythm in the mouse retina. Spaceflight missions often expose astronauts to atypical sleep–wake cycles and work schedules [50,51]. Changes in circadian rhythm may have a significant impact on neurobehavior and neurophysiological processes [52]. Previous studies have found that circadian rhythm disruption or circadian misalignment in astronauts may affect performance and cognitive function [53,54,55]. Our results suggest that changes in circadian rhythm induced by spaceflight may involve the DG region. This could help in developing countermeasures for sleep disturbance during spaceflight.

Our findings will help to improve the understanding of regional variation or sensitivities in susceptibility to brain injury and neurodegenerative diseases that exist during spaceflight. In addition, our findings may provide novel insight into cellular mechanisms and operational risks that underlie the effects of spaceflight-mediated structural and functional damage to different brain regions. In future studies, the regulatory mechanism for regional differences in stress response should be further explored in terms of variations of cell types, vasculature, neurotransmitter profiles, hemodynamics, and metabolism.

Studies have shown a spaceflight-induced increase in the production of lipid peroxidation products and a decrease in antioxidant enzyme activity [26]. It has been suggested that in stressful environments, antioxidant expression of SOD and catalase are reduced to conserve energy [56]. BuOE, a recently developed antioxidant compound, is among the most highly potent metalloporphyrins that have been evaluated for safety and efficacy [57]. Many animal studies have demonstrated that BuOE is highly effective in mitigating oxidative stress induced by radiation exposure [58]. We therefore treated a subset of our mice with BuOE in order to test its ability to attenuate spaceflight-induced changes in transcription in the CA, DG, FCT, and CT. Treatment with BuOE reversed spaceflight-induced changes in transcription of a substantial number of DEGs, and a statistically significant number of spaceflight-induced DEGs changed in the opposite direction as did BuOE-induced DEGs, suggesting that BuOE may be effective in attenuating at least some of the effects of spaceflight stress on the brain.

We presented evidence that BuOE may attenuate the consequences of oxidative stress on the brain during spaceflight. BuOE attenuated spaceflight-induced changes in the expression of a number of genes in the oxidative stress–responsive pathways that were enriched due to spaceflight. We note that BuOE had a particularly strong effect in reversing spaceflight-induced changes of the following genes in oxidative stress–responsive pathways (Figure 6): *Prkca* in the CA, *Atp2b4* in the DG, *Homer1* in the FCT, and *Itga7* in the CT. Since we had identified known associations between spaceflight-induced DEGs and neurological diseases, we speculated whether BuOE attenuated the effects of these genes as well. Indeed, we found that BuOE reversed spaceflight-induced changes in the expression of several genes with known associations to Alzheimer’s disease.

Our results suggest that BuOE affects each brain region in a distinct way. The PCA (Figure 5A) suggests that the region with the greatest degree of attenuation by BuOE was the CT, in that treatment with BuOE brings the spaceflight transcriptomic profile closest to that of grounded controls in CT. On the other hand, BuOE treatment actually moved the spaceflight transcriptomic profile further away from grounded control animals in FCT, suggesting that FCT may be the region with the most resistance to superoxide scavengers. The DG and CA had intermediate responses to BuOE relative to the CT and FCT. Taken together, our results on BuOE warrant further study as a potential therapeutic candidate for CNS protection against brain injury and neurodegeneration induced by ionizing radiation and environmental stress.

## 4. Materials and Methods

### 4.1. Spaceflight and Mouse Groups

Ten-week-old C57BL/6 male mice were launched at the Kennedy Space Center (KSC) and spent 35 days aboard the International Space Station (ISS). All mice were maintained at an ambient temperature of 26–28 °C with a 12-h light/dark cycle during the flight. All mice were provided NASA Nutrient-upgraded Rodent Food Bar (NuRFB) and autoclaved deionized water ad libitum. MnTnBuOE-2-PyP5+ (BuOE) at 1 mg/kg (0.2 mL) was administrated subcutaneously 7 days prior to the flight launch and weekly aboard the ISS. All mice were subdivided into saline or BuOE-treated groups. Upon return to the Earth, spaceflight mice were transported to the research laboratory at Roskamp Institute, Sarasota, Florida, within 20 h of splashdown. Mice were exsanguinated by closed-cardiac blood collection under deep Ketamine/Xylazine (150/45 mg/kg) anesthesia, followed by cervical dislocation as a secondary euthanasia method to ensure death. Ground control mice were maintained on Earth for 35 days in flight hardware cages under similar environmental conditions as the flight groups, including the same food, light/dark, temperature, treatment (SAL or BuOE), and euthanasia regimens. The protocol for GC mice commenced three days subsequent to the commencement of the protocol for spaceflight mice.

After sacrifice, mouse brains were then removed and prepared as follows. The left hemibrains were fixed in 4% paraformaldehyde in phosphate-buffered saline (PBS) for 24 h and then rinsed and washed with PBS for immunohistochemistry (IHC) assays or spatial genomics profiling. The right brains were flash-frozen and stored at −80 °C for further analysis. The study was approved by the Institutional Animal Care and Use Committee (IACUC) of Loma Linda University (LLU), Roskamp Institute, and The National Aeronautics and Space Administration (NASA).

### 4.2. GeoMx DSP

DSP technology enables us to select specific regions of interest (ROIs) with high magnification [59,60]. Three 6 μm formalin-fixed, paraffin-embedded (FFPE) tissue sections selected at a similar focal plane from three mice in each group were mounted on one slide for a total of 4 slides, one for each group (GC-SAL, GC-BuOE, FLT-SAL, and FLT-BuOE). Slides were baked for 2 h at 65 °C for paraffin removal before loading onto a Leica BOND RX for tissue rehydration in EtOH and ddH2O, heat-induced epitope retrieval (ER2 for 20 min at 100 °C) and proteinase K treatment (1.0 μg/mL for 15 min at 37 °C). Tissue sections were then hybridized with the Mouse Whole Transcriptome Atlas (WTA) probes overnight at 37 °C. Following 2 × 5 min stringent washes (1:1 4× SSC buffer and formamide), the slides were blocked for 30 min and then incubated with morphology marker antibodies for 1 h to guide region of interest (ROI) selection: GFAP (Texas Red/615nm channel, Alexa 594 fluorophore, 1:200 dilution, shown in green in Figure 1A, NBP2-33184DL594, Novus Biologicals, Littleton, CO, USA), and Iba1 (Cy5/666nm channel, Alexa 647 fluorophore, 1:100 dilution, shown in yellow in Figure 1A, 48934S, Cell Signalling Technologies, Danvers, MA, USA). Syto83 (Cy3/568 nm channel, Alexa 532 fluorophore, 1:10 dilution, shown in blue in Figure 1A, S11364, Invitrogen, Waltham, MA, USA) was used as a nuclear stain.

After ROI selection, UV light was directed by the GeoMx (https://nanostring.com/products/geomx-digital-spatial-profiler/, accessed on 3 February 2022, NanoString Technologies, Inc., Seattle, WA, USA) device at each area of illumination (AOI), releasing the RNA-ID containing oligonucleotide tags from the WTA probes for collection into a unique well for each AOI. For library preparation, Illumina i5 and i7 dual indexing primers were added to the oligonucleotide tags during PCR to uniquely index each AOI. AMPure XP beads (Beckman Coulter, Indianapolis, IN, USA) were used for PCR purification. Library concentration was measured using a Qubit fluorometer (Thermo Fisher Scientific, Waltham, MA, USA), and quality was assessed using a Bioanalyzer (Agilent Technologies Inc., Santa Clara, CA, USA). Sequencing was performed on an Illumina NovaSeq 6000 (Illumina Inc., San Diego, CA, USA).

### 4.3. GeoMx DSP Data Analysis

Raw .fastq files were processed into gene count data for each AOI using the GeoMx^®^ NGS Pipeline via Amazon Web Services. All subsequent data analysis described in this subsection was performed using the online GeoMx DSP analysis platform [61], GeoMx DSP Control Center version 3.0.0.111. Raw reads were trimmed, stitched, aligned, and finally deduplicated using default parameters. Using Q3 normalized read count data, a differential expression analysis of FLT-SAL versus GC-SAL, and FLT-BuOE versus FLT-GC, was performed for each brain region independently. Statistical significance of differential expression was measured by *t*-test (non-paired), and permutation q-values (*p*-adjusted) were calculated. Genes with q < 0.05 and |log_2_ FC| > 0.585 were considered differentially expressed and used for downstream analysis.

### 4.4. Association of DEG’s with Neurological Disease

A list of genes with known associations to human neurological diseases was obtained using the DisGeNET [62] browse feature with the filters “Disease class: Mental disorders” and “Semantic type: disease or syndrome”. Only genes from curated databases were used.

### 4.5. Pathway Analysis

The online tool Enrichr [63,64,65] was used to perform pathway mapping for GC-SAL vs. FLT-SAL DEGs from each region independently. Enriched pathways in this paper are from the KEGG [65] 2019 Mouse pathway analysis in the “legacy” tab of the Enrichr website.

### 4.6. Principal Component Analysis

Q3 normalized read counts output by the online GeoMx DSP analysis platform, as described above, were used as inputs for the PCA. The analysis was done in R version 4.0.3. The R function aov() [66] was used to perform a linear analysis of variance (ANOVA) at each gene, using each biological sample as a factor. The R function qvalue() [67] was then used to calculate ANOVA q-values for all genes, based on the *p*-values output by aov. Significant genes from the ANOVA (q < 0.05) were then input into the R function PCA() [68] in order to generate PCA plots.

### 4.7. Statistical Comparison of Spaceflight-Induced and BuOE-Induced DEG Overlaps

For a given brain region, let N = total # of spaceflight-induced DEGs (GC-SAL vs. FLT-SAL) plus total # of BuOE-induced DEGs (FLT-SAL vs. FLT-BuOE) for that region. For each brain region, a 2 × 2 contingency matrix,

X_11_ X_12_

X_21_ X_22_,

was constructed, where X_11_ = # of spaceflight-induced DEGs changing in the opposite direction as BuOE-induced DEGs, X_12_ = N − X_11_, X_21_ = # of spaceflight-induced DEGs changing in the same direction as BuOE-induced DEGs, X_22_ = N − X_21_. Fisher’s exact test was then calculated on each 2 × 2 contingency matrix in order to obtain a *p*-value for the null hypothesis that the proportion of overlapping DEGs changing in the opposite direction was the same as the proportion of overlapping DEGs changing in the same direction.

### 4.8. Permutation Testing of Spaceflight-Induced and BuOE-Induced DEG Overlaps

For each brain region, a random set of BuOE-induced DEGs was selected as follows. The total set of gene symbols analyzed was shuffled using the “shuf” command in Ubuntu 20.04.5 LTS. The top N_up_ genes in the shuffled file were selected as the upregulated, BuOE-induced DEGs, where N_up_ = total # of actual upregulated, BuOE-induced DEGs (FLT-SAL vs. FLT-BuOE) for the brain region. Similarly, the bottom N_down_ genes in the shuffled file were selected as the downregulated, BuOE-induced DEGs, where N_down_ = total # of actual downregulated, BuOE-induced DEGs in the brain region. The randomly selected DEGs were then compared with the actual spaceflight-induced DEGs (GC-SAL vs. FLT-SAL) to determine how many changed in opposite directions. This shuffling procedure was repeated 1000 times for each brain region.

## Data Availability

The data presented in this study are openly available in NCBI GEO, accession number GSE239336. Custom scripts used for data analysis in this study are available on the github repository (https://github.com/ikremsky/Scripts-for-Kremsky-et-al.-GeoMx-DSP-mouse-spaceflight-study, accessed on 21 July 2023).

## Supplementary Materials

The following supporting information can be downloaded at: https://www.mdpi.com/article/10.3390/ijms241713569/s1.
