# Supplementary material for: Spaceflight-Induced Gene Expression Profiles in the Mouse Brain Are Attenuated by Treatment with the Antioxidant BuOE
Source: Int J Mol Sci. 2023 Sep 1;24(17):13569. doi: 10.3390/ijms241713569 (PMC10487739; doi:10.3390/ijms241713569)
Supplement: Supplementary file 1 [file ijms-24-13569-s001.zip › Supplementary File S1.pdf]

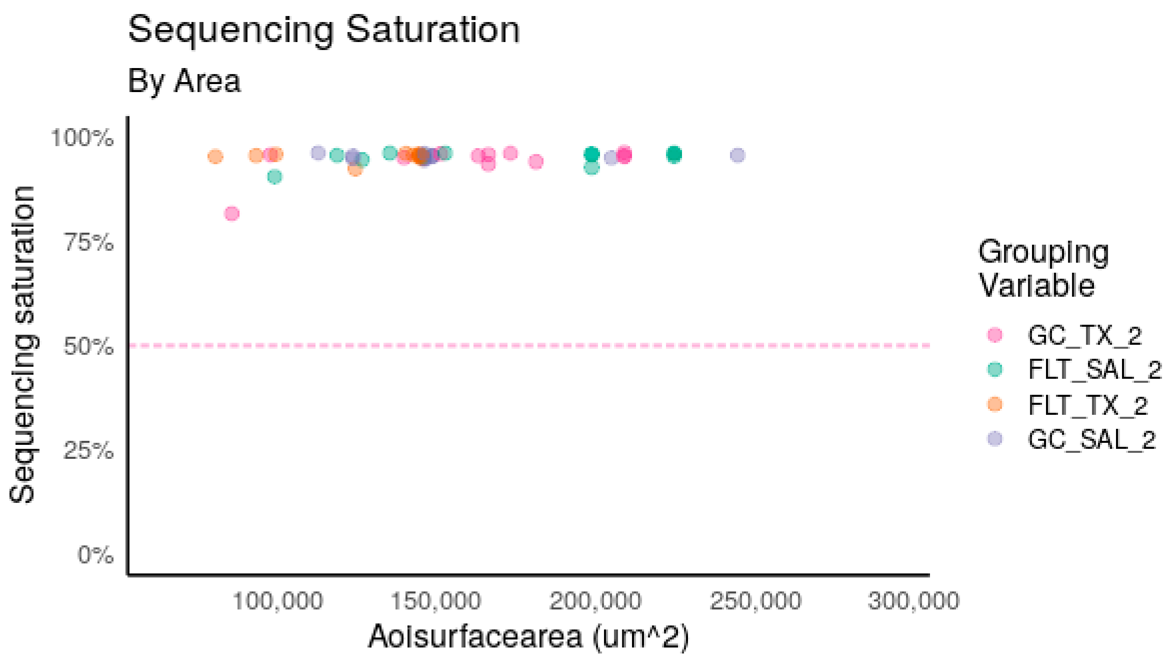

Sequencing Saturation
By Area
Sequencing saturation
100%
75%
50%
25%
0%
100,000
150,000
200,000
250,000
300,000
Aoisurfacearea (um^2)
Grouping Variable
GC_TX_2
FLT_SAL_2
FLT_TX_2
GC_SAL_2

(A)

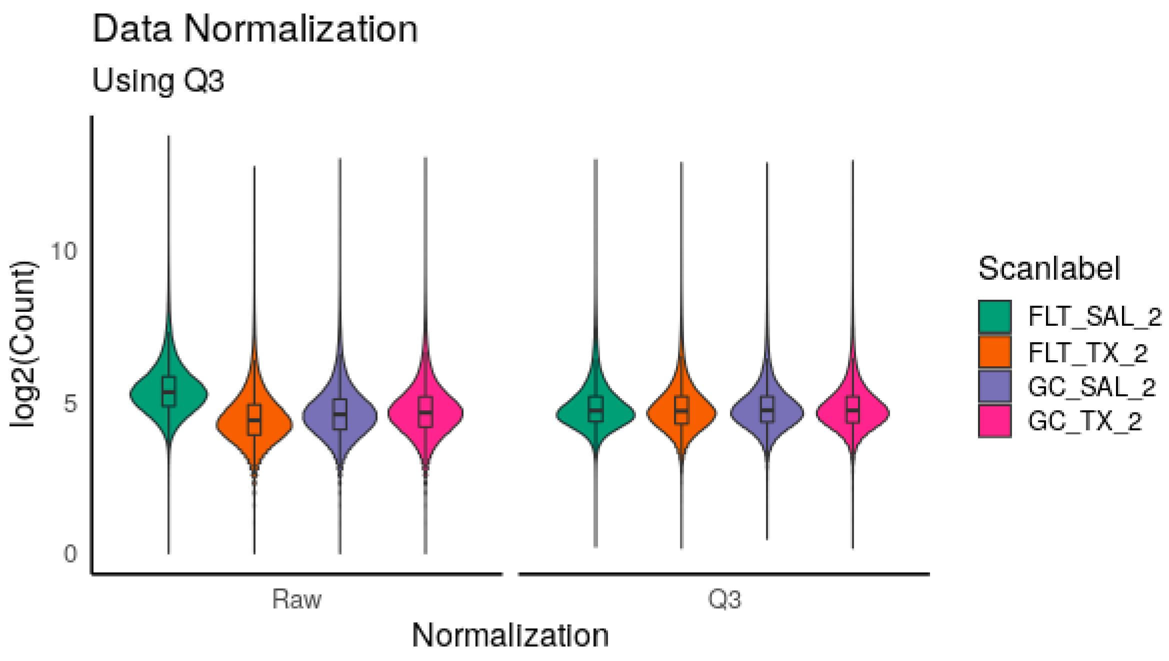

Data Normalization
Using Q3
log2(Count)
10
5
0
Raw
Q3
Normalization
Scanlabel
FLT_SAL_2
FLT_TX_2
GC_SAL_2
GC_TX_2

(B)

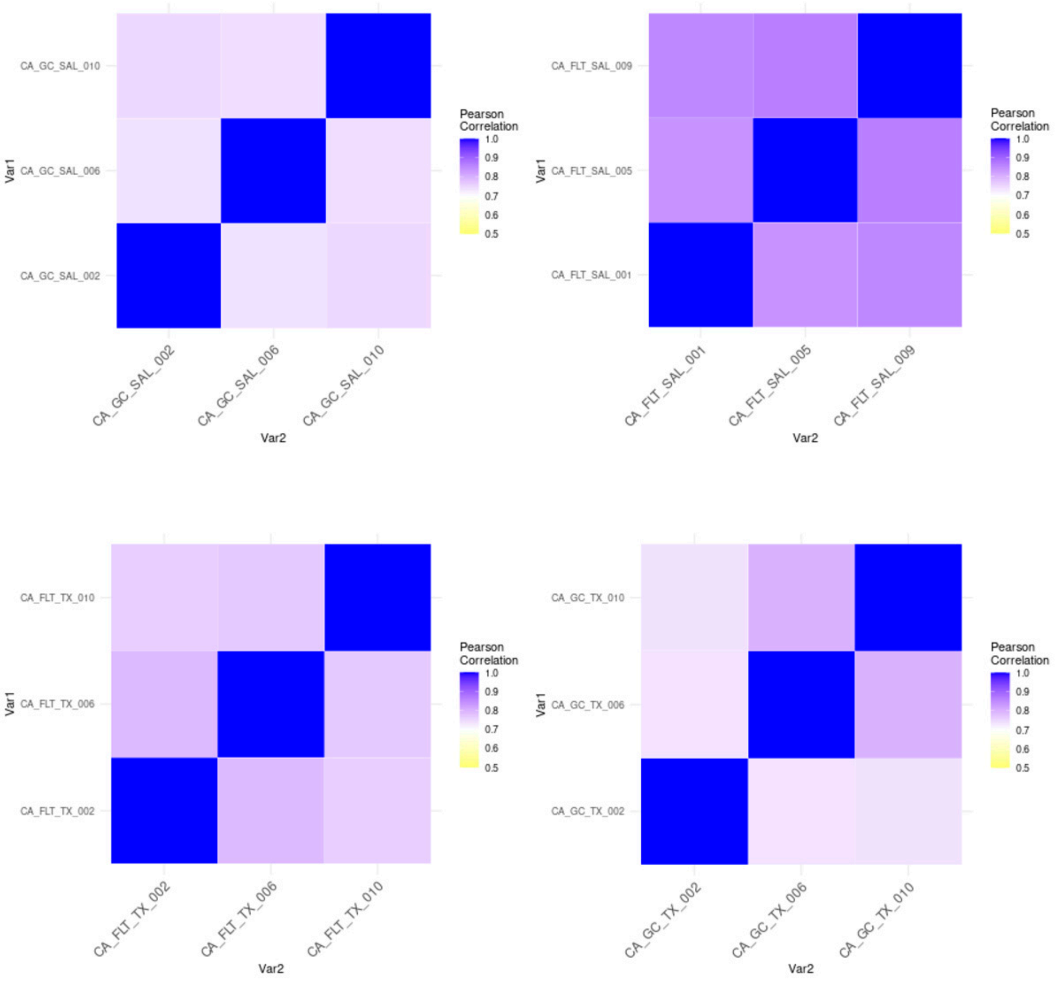

(C) Replicate correlations CA (TX=BuOE)

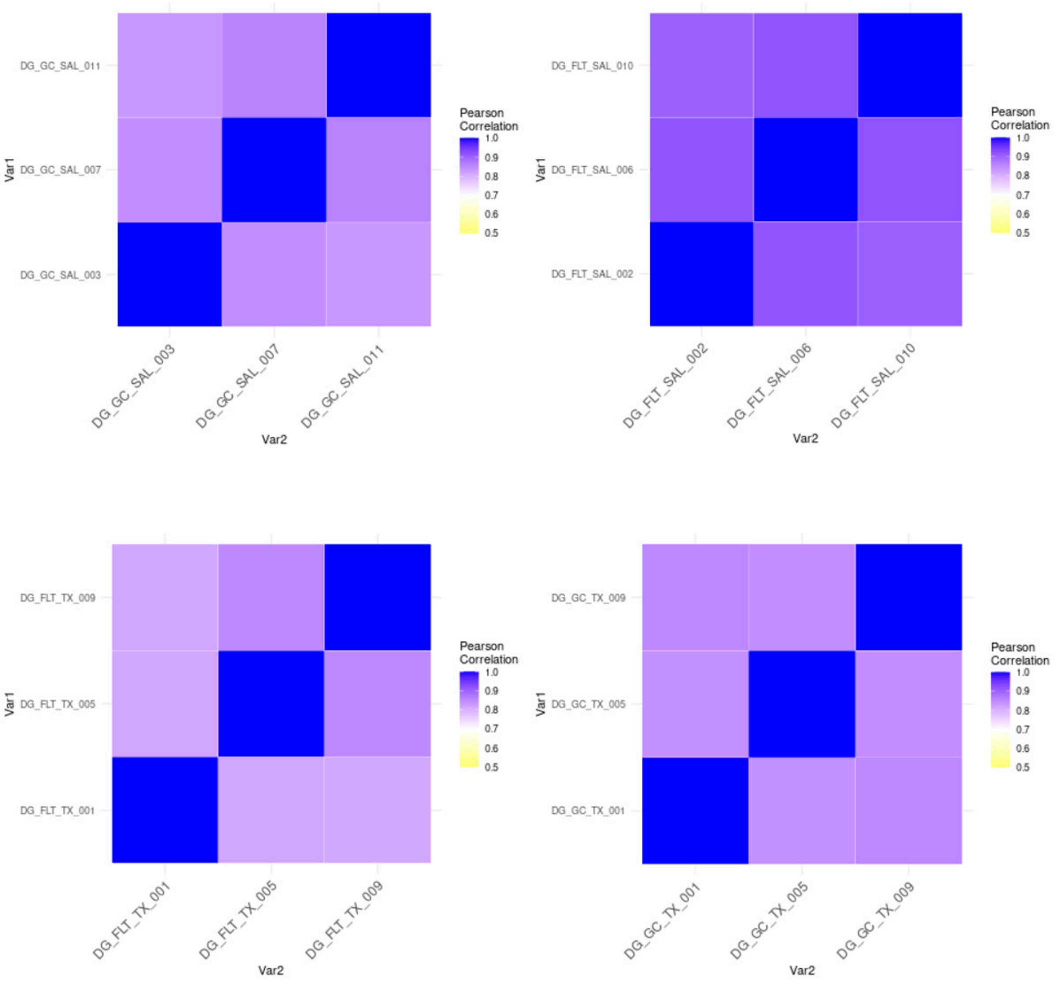

(D) Replicate correlations DG (TX=BuOE)

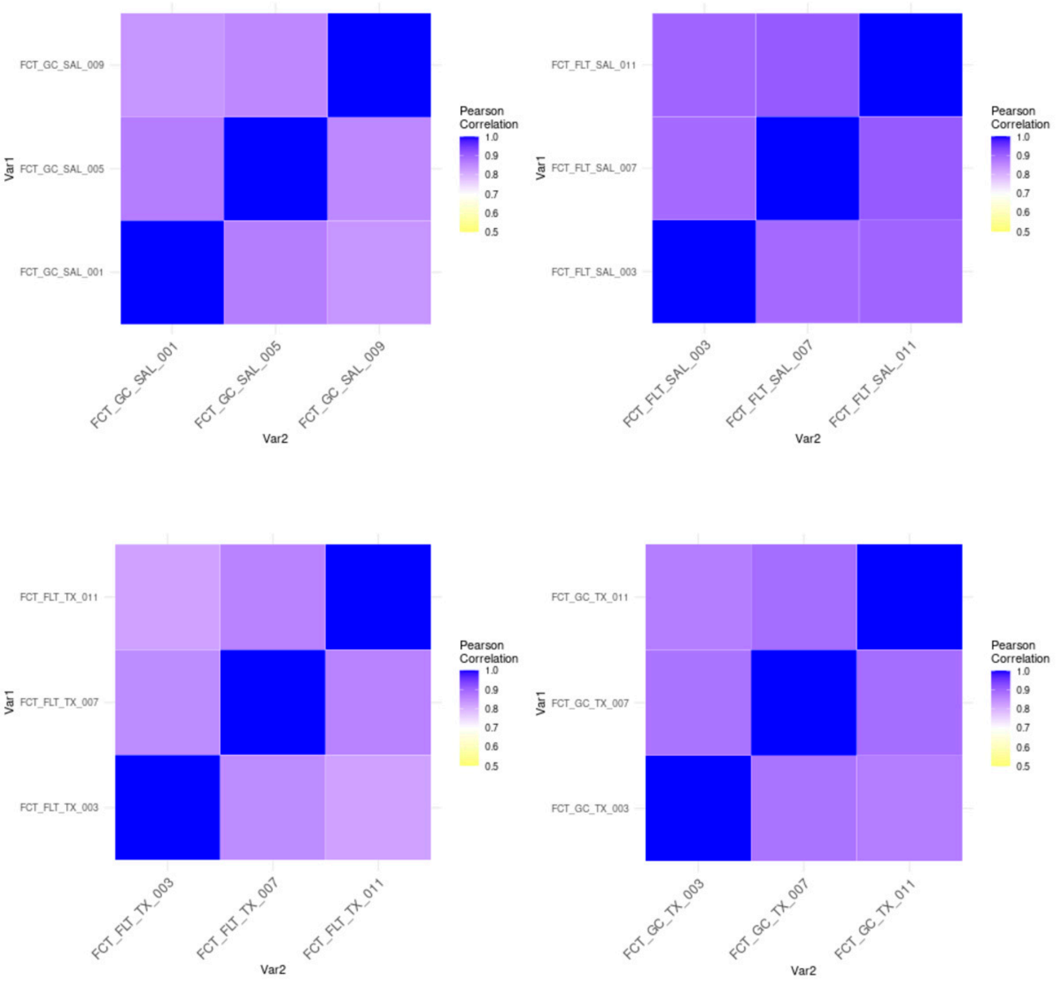

(E) Replicate correlations FCT (TX=BuOE)

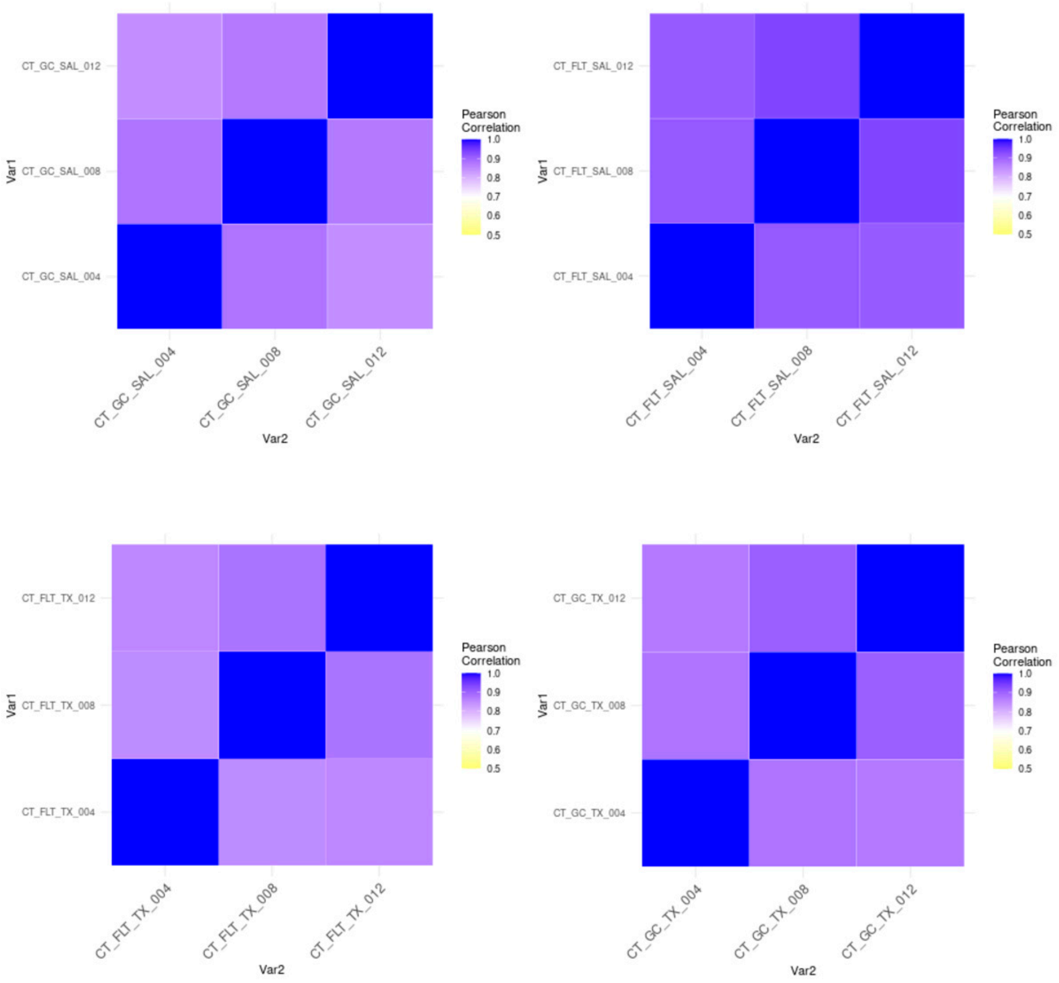

(F) Replicate correlations CT (TX=BuOE)

Figure S1. Validation of DSP data. A Plot of sequencing saturation, as generated by the online GeoMx DSP Control Center. **B** Violin plots showing the distributions of raw and normalized read counts for each sample, as provided by Nanostring. **C-F** Heatmaps showing Pearson's correlation coefficient when comparing log-transformed, normalized gene expression values (ln(normalized expression + 1)) between replicates of the indicated samples and regions.

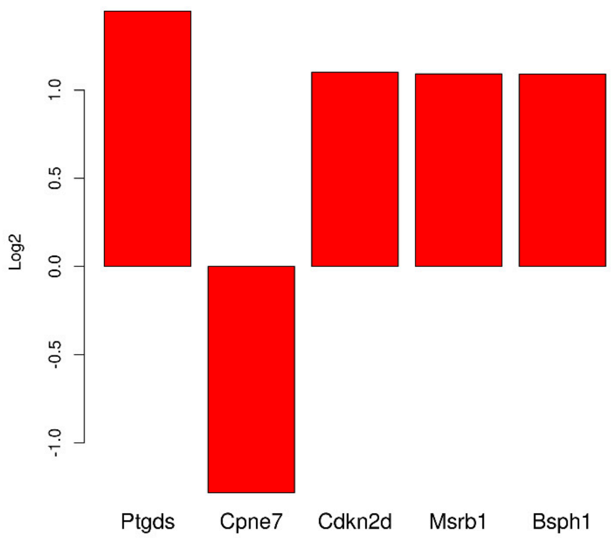
Log2
1.0
0.5
0.0
-0.5
-1.0
Ptgds
Cpne7
Cdkn2d
Msrb1
Bsph1

(A) CA

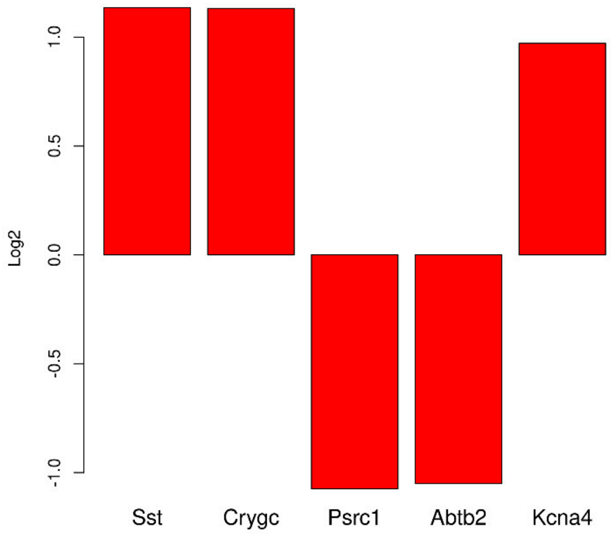
Log2
1.0
0.5
0.0
-0.5
-1.0
Sst
Crygc
Psrc1
Abtb2
Kcna4

(B) DG

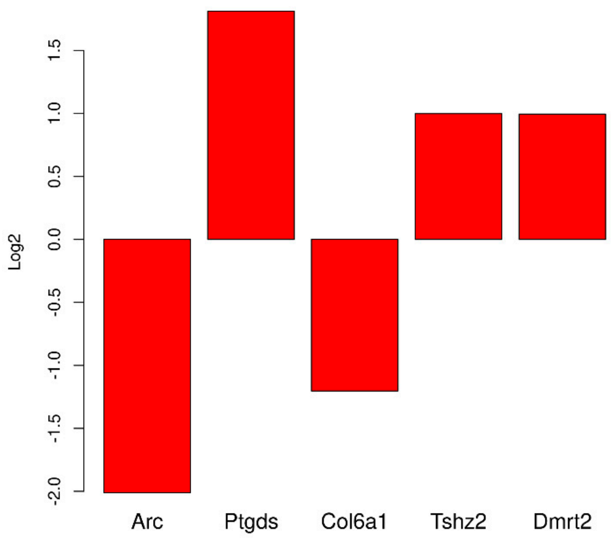

(C) FCT

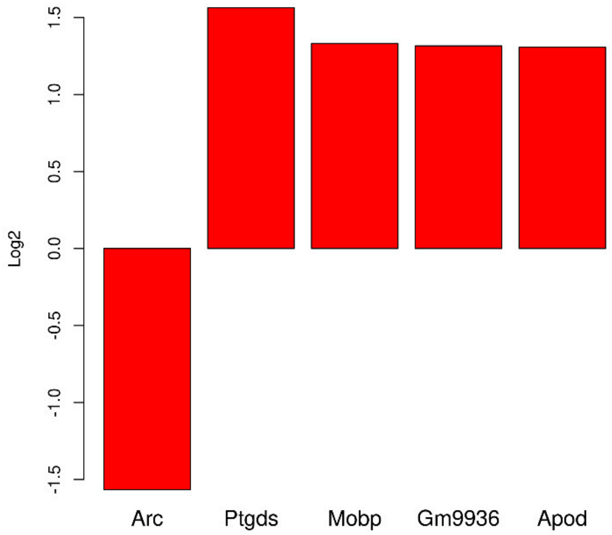

(D) CT

Figure S2. Related to Figure 3. A-D Barplots showing log_2 Fold Change values for the top 5 DEGs by magnitude of log_2 Fold Change for the indicated brain regions.

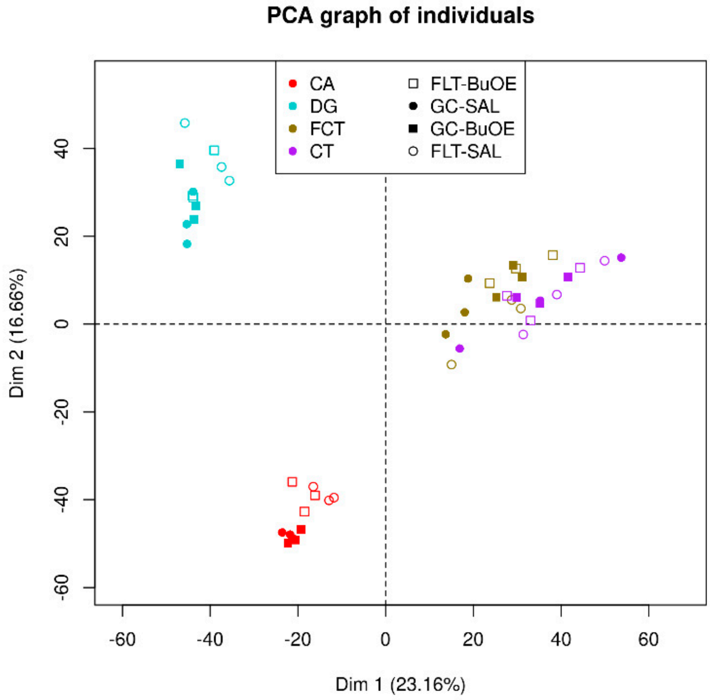
PCA graph of individuals
CA
DG
FCT
CT
FLT-BuOE
GC-SAL
GC-BuOE
FLT-SAL
Dim 2 (16.66%)
Dim 1 (23.16%)
-60
-40
-20
0
20
40
60

(A)

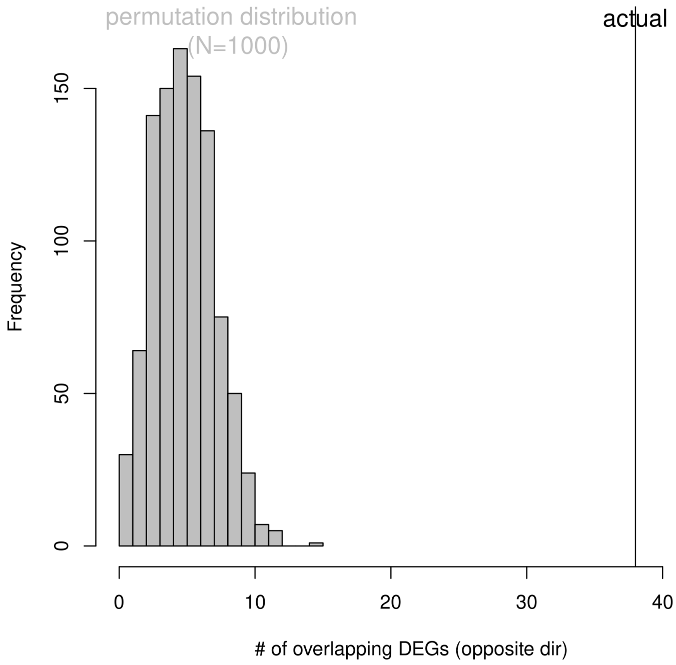
permutation distribution
(N=1000)
actual
Frequency
0
50
100
150
0
10
20
30
40
# of overlapping DEGs (opposite dir)

(B) CA

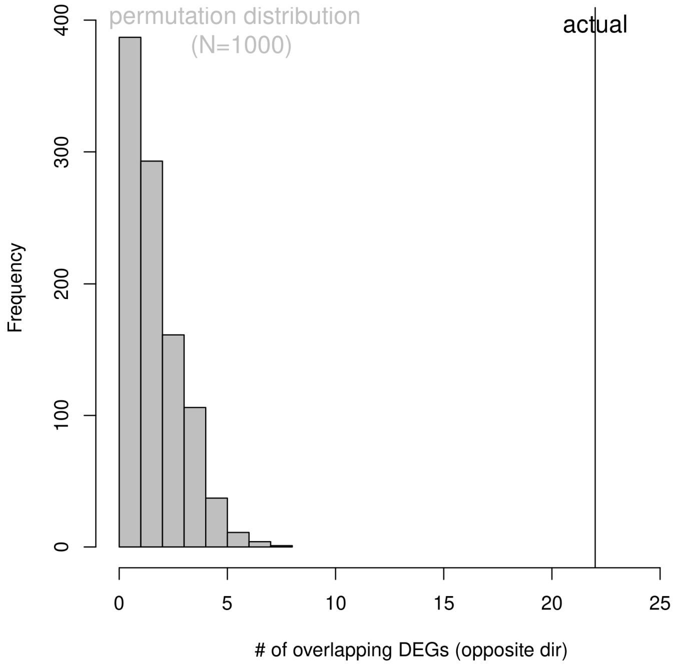
permutation distribution
(N=1000)
actual
Frequency
0
100
200
300
400
0
5
10
15
20
25
# of overlapping DEGs (opposite dir)

(C) DG

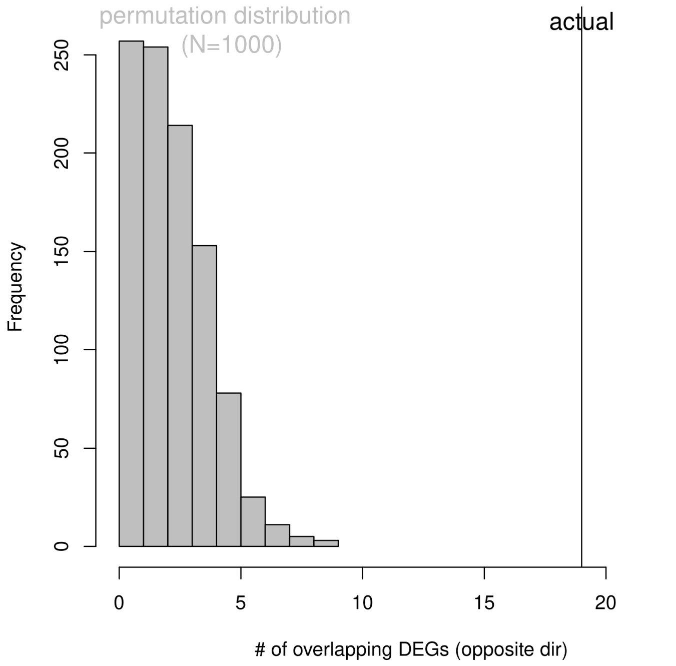
permutation distribution
(N=1000)
actual
Frequency
0
50
100
150
200
250
0
5
10
15
20
# of overlapping DEGs (opposite dir)

(D) FCT

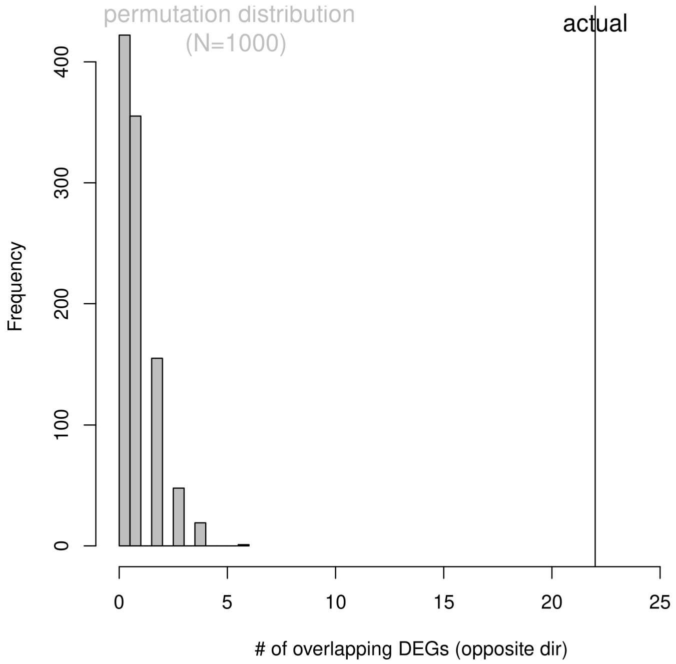

(E) CT

Figure S3. Related to Figure 5. A PCA of data from the indicated samples, with all individual replicates treated separately. **B-E** Histograms showing the distribution of the number of DEGs that change in opposite directions between GC-SAL vs. FLT-SAL and FLT-SAL vs. FLT-BuOE for the indicated regions, when randomly shuffling to select DEGs for FLT-SAL vs. FLT-BuOE. The vertical line indicates the actual number of DEGs that change in opposite directions for the two comparisons. N=1000 permutations were performed when randomly selecting FLT-SAL vs. FLT-BuOE DEGs.

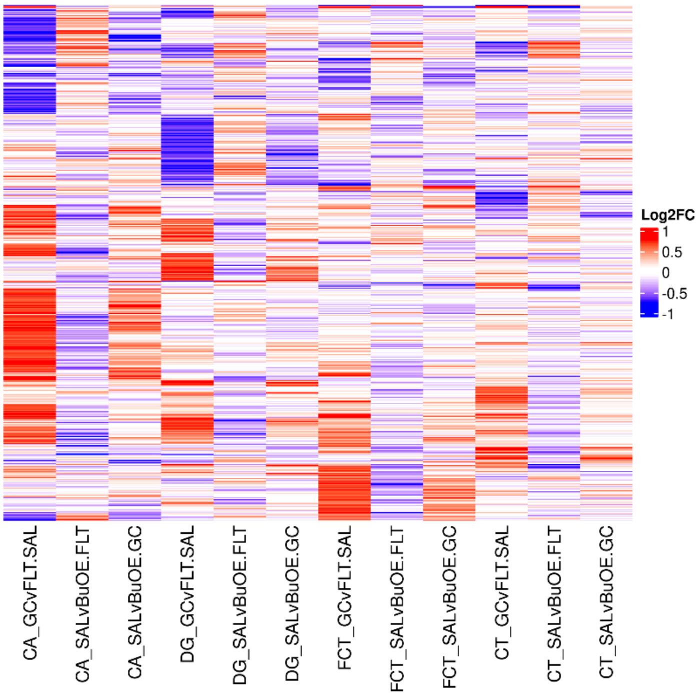

(A)

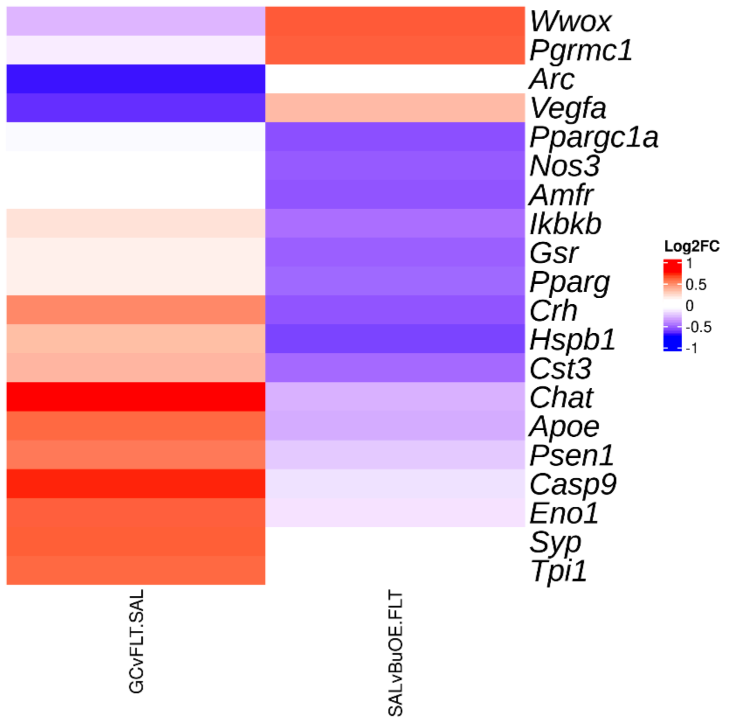

(B) AD genes in CA

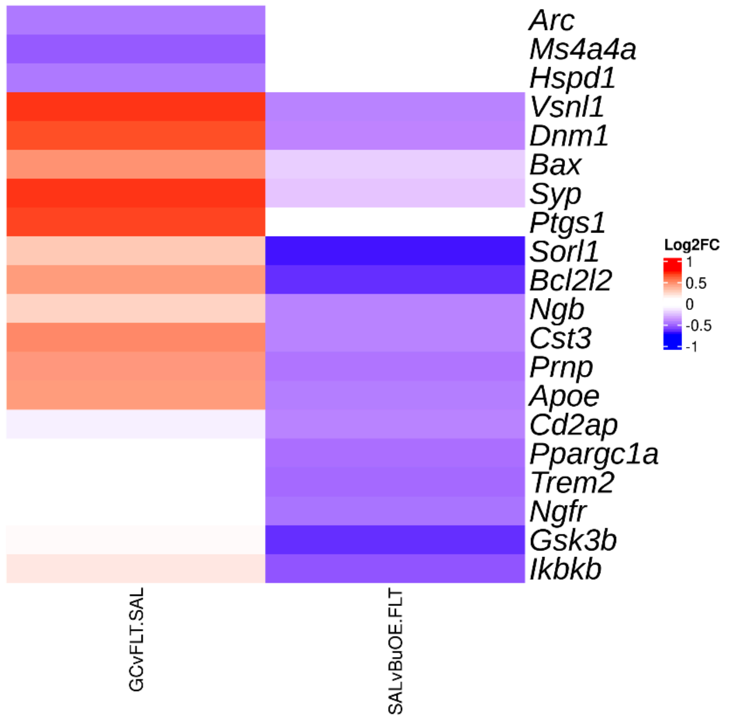

(C) AD genes in DG

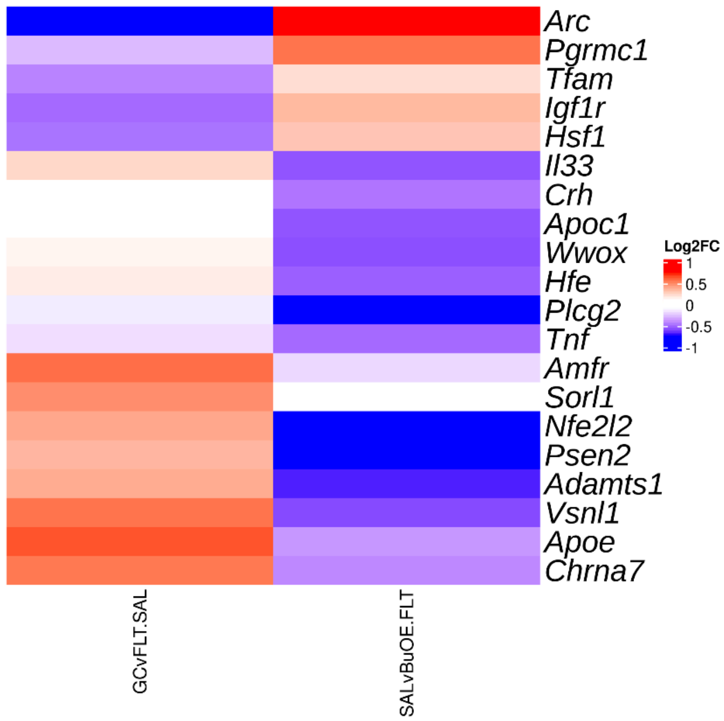

(D) AD genes in FCT

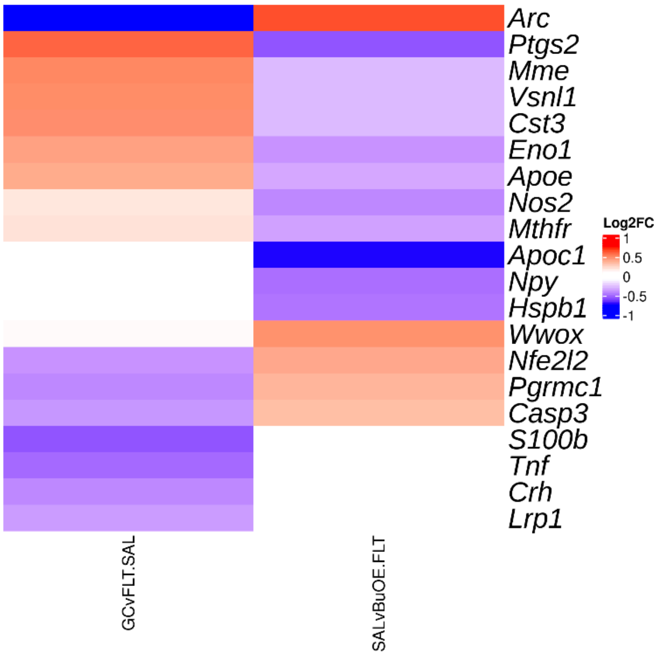

(E) AD genes in CT

Figure S4. Related to Figure 6. A Heatmap of $\log_2$ Fold Change (FC) values from the indicated comparisons and brain regions, at the GC-SAL vs. FLT-SAL DEGs. Genes are ordered by hierarchical clustering. **B-E** Heatmaps of $\log_2$ FC values from the indicated comparisons, at genes associated with Alzheimer's Disease, at the indicated brain regions. For each brain region, the top 20 genes, ordered by the maximum of the $\log_2$ FC from both comparisons, are shown. Genes are ordered by hierarchical clustering. GCvFLT.SAL = GC-SAL vs. FLT-SAL; SALvBuOE.FLT = FLT-SAL vs. FLT- BuOE; SALvBuOE.GC = GC-SAL vs. GC-BuOE.
